# Three-dimensional tumor model mimics stromal – breast cancer cells signaling

**DOI:** 10.18632/oncotarget.22922

**Published:** 2017-12-05

**Authors:** Stephanie Lemmo Ham, Pradip Shahi Thakuri, Madison Plaster, Jun Li, Kathryn E. Luker, Gary D. Luker, Hossein Tavana

**Affiliations:** ^1^ Department of Biomedical Engineering, The University of Akron, Akron, OH 44325, USA; ^2^ Department of Mathematical Sciences, Kent State University, Kent, OH 44242, USA; ^3^ Department of Radiology, Microbiology and Immunology, Biomedical Engineering, University of Michigan, Ann Arbor, MI 48109, USA

**Keywords:** TNBC, tumor-stromal signaling, CXCL12, CXCR4, three-dimensional culture

## Abstract

Tumor stroma is a major contributor to the biological aggressiveness of cancer cells. Cancer cells induce activation of normal fibroblasts to carcinoma-associated fibroblasts (CAFs), which promote survival, proliferation, metastasis, and drug resistance of cancer cells. A better understanding of these interactions could lead to new, targeted therapies for cancers with limited treatment options, such as triple negative breast cancer (TNBC). To overcome limitations of standard monolayer cell cultures and xenograft models that lack tumor complexity and/or human stroma, we have developed a high throughput tumor spheroid technology utilizing a polymeric aqueous two-phase system to conveniently model interactions of CAFs and TNBC cells and quantify effects on signaling and drug resistance of cancer cells. We focused on signaling by chemokine CXCL12, a hallmark molecule secreted by CAFs, and receptor CXCR4, a driver of tumor progression and metastasis in TNBC. Using three-dimensional stromal-TNBC cells cultures, we demonstrate that CXCL12 – CXCR4 signaling significantly increases growth of TNBC cells and drug resistance through activation of mitogen-activated protein kinase (MAPK) and phosphoinositide 3-kinase (PI3K) pathways. Despite resistance to standard chemotherapy, upregulation of MAPK and PI3K signaling sensitizes TNBC cells in co-culture spheroids to specific inhibitors of these kinase pathways. Furthermore, disrupting CXCL12 – CXCR4 signaling diminishes drug resistance of TNBC cells in co-culture spheroid models. This work illustrates the capability to identify mechanisms of drug resistance and overcome them using our engineered model of tumor-stromal interactions.

## INTRODUCTION

Signaling between cancer cells and tumor stroma drives all stages of cancer initiation and progression [1–4]. Histological examinations of human tumors show greater stromal content in more advanced and larger tumors, and high content of stromal cells correlates with greater risk of relapse and reduced survival [5–8]. Fibroblasts are the most abundant stromal cell type in epithelial tumors [9–12]. Cancer cells dynamically activate surrounding fibroblasts to produce growth factors, hormones, and cytokines that fuel tumor growth [13], and facilitate progression to metastasis [14]. These activated fibroblasts, termed carcinoma-associated fibroblasts (CAFs), regulate cancer cells through paracrine signaling and direct intercellular interactions. In a study of breast carcinoma, about 80 percent of fibroblasts exhibited an activated phenotype [15].

Production of the chemokine CXCL12 (also known as stromal cell-derived factor-1) is a hallmark feature of CAFs [16]. CXCL12 signals through CXCR4 and CXCR7 receptors upregulated on cancer cells [17], and activates multiple molecular pathways such as the mitogen-activated protein kinase (MAPK), inositol 1,4,5-triphosphate (IP3), and phosphoinositide 3-kinase (PI3K) [18]. Signaling through these pathways promotes survival, proliferation, metastasis, and drug resistance of cancer cells [18–21]. The CXCL12 – CXCR4/CXCR7 signaling axis is highly active in breast cancers [22, 23]. Analysis of human breast tumors showed that among the different molecular subtypes, triple negative breast cancer (TNBC) cells have the most elevated expression of CXCR4 [24]. Considering the lack of targeted therapies for TNBC [25], disrupting this chemokine signaling may offer a potential new therapeutic approach. In addition, investigating pathways downstream of this chemokine signaling axis in TNBC cells potentially will unveil new molecular targets for therapy.

Studies of interactions among stroma and cancer cells typically use monolayer cell cultures or xenograft models. Monolayer cultures lack the complexity of tumors including a compact three-dimensional morphology, close intercellular contacts, exposure of cells to gradients of soluble factors and oxygen, and non-uniform, spatially-dependent cell proliferation [26–28]. Thus, cells in monolayer cultures often fail to reproduce key characteristics of tumors. Xenografts present a physiological system for cancer research. However, they are limited in terms of absence of human tumor stroma, failure of some murine cytokines to activate human receptors, disparities in dynamics of tumor growth and progression compared to human tumors, and need for complex tools and biological assays to study stromal effects on cancer cells [29–31]. Since the introduction of tissue microarrays and next generation sequencing [32], these technologies have been used for high throughput assessment of biomarkers in thousands of tumor samples from biopsies using standard assays such as immunohistochemistry and fluorescent in-situ hybridization [33]. These technologies are an invaluable tool in clinical oncology to develop diagnostic tests and identify disease biomarkers. Nevertheless, tissue microarrays and similar biopsy-based assays for endpoint analysis of fixed samples do not capture dynamic tumor-stromal interactions in tumor microenvironments. Tumors constantly transform both spatially and temporally. Interactions between stromal and cancer cells play a major role in conferring functional changes to cancer cells such as gain of cancer stem cell and mesenchymal characteristics, altered metabolism, drug resistance, migration and invasion, and survival [34]. Mechanistic understanding of these complex events in tumors requires approaches that reproduce dynamic signaling of cancer and stromal cells as it occurs in native tissues.

Three-dimensional (3D) cultures of cells as spheroids provide relevant *in vitro* tumor models to recapitulate architecture and complex intercellular network of tumors and emulate stromal-cancer cells interactions [26, 35–37]. We recently developed a robotic, high throughput spheroid microprinting technology to mass produce homogenously-sized spheroids that exhibit key biology properties of solid tumors [38–41]. Here, we utilized this technology and formed an array of co-culture spheroids of TNBC and stromal cells to examine CXCL12 signaling through CXCR4 and CXCR7 receptors on TNBC cells. Using different cellular assays and molecular analyses, we demonstrated that CXCL12 – CXCR4 signaling significantly increases spheroid proliferation and TNBC cell growth. This signaling conferred resistance to standard chemotherapy drug treatment through activation of MAPK and PI3K pathways. We found that CXCL12 – CXCR4 signaling induces sensitivity of the cancer cells to specific molecular inhibitors of MAPK and PI3K pathways, preventing proliferation of TNBC cells. This work establishes the feasibility of studying tumor-stromal interactions using our engineered solid tumor models and offers a convenient preclinical tool to identify new treatment approaches.

## RESULTS AND DISCUSSION

### Aqueous two-phase system (ATPS) microprinting of TNBC-stromal cells co-culture spheroids

**Figure 1:**
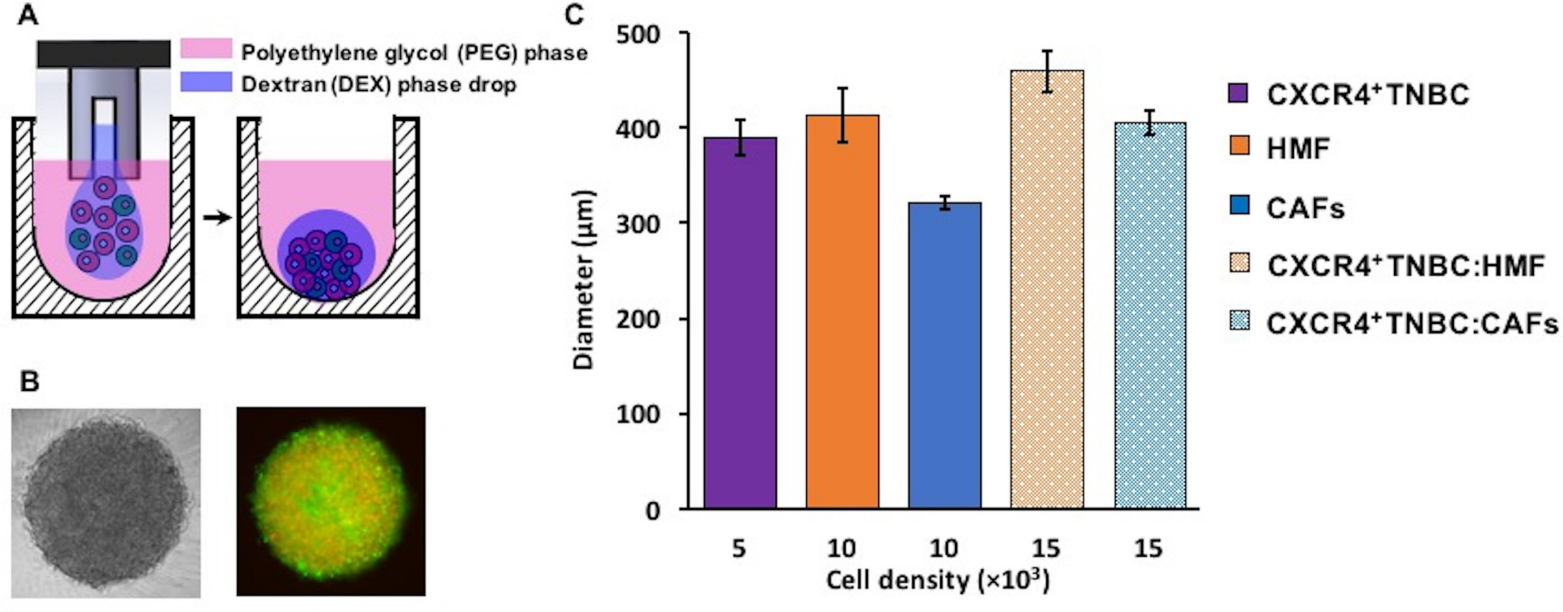
(**A–B**) Cancer cells remain confined in the 0.3 µl DEX phase drop (purple) suspended in the immiscible immersion PEG phase (pink) and autonomously aggregate to form a co-culture spheroid of triple negative breast cancer cells (green) and human mammary fibroblasts (red) in 48 hrs. Colors in panel (A) are for presentation purpose only. (**C**) Resulting spheroids of different co-culture models are consistently sized with low standard errors.

The ATPS technology facilitates partitioning of cancer and stromal cells to the DEX phase nanodrop to spontaneously form a mono-culture or a co-culture spheroid within 24–48 hours of incubation (Figure 1A–1B) [42, 43]. Importantly, nutrients and waste products of cells freely diffuse between the DEX phase nanodrop and the immersion PEG phase [38]. Adapting the technology to robotics enabled formation of spheroids in standard 384-microwell plates [44]. For co-culture spheroids, we selected a ratio of 1:2 TNBC to stromal cells and a total cell density of 1.5 × 10^4^ cells/0.3 μl of DEX phase drop. This ratio was to mimic more advanced and larger human breast tumors that have greater stromal content than cancer cells [5, 6, 45, 46]. Using larger ratios of 1:3 and 1:4 (breast cancer cells to fibroblasts) while keeping the initial breast cancer cell density constant at 5 × 10^3^ cells per DEX phase drop did not alter growth of TNBC cells (Supplementary Figure 1), consistent with other studies [47, 48]. This microprinting approach gave consistently-sized mono-culture spheroids of CXCR4^+^TNBC cells (5 × 10^3^ cells), mono-culture spheroids of fibroblast cells, HMF and CAFs, (1 × 10^4^ cells), and co-culture spheroids of CXCR4^+^TNBC cells with HMF cells or CAFs (1.5 × 10^4^ cells with a 1:2 TNBC to stromal cells ratio) (Figure 1C). The spheroid size consistency was measured from two separate experiments to ensure that spheroids of each model had a similar initial metabolic activity baseline. Importantly, the 1.5 × 10^4^ cell density co-culture spheroids containing HMF cells or CAFs were not statistically different in size (*p* > 0.05), eliminating potential effects of size differences of the spheroids on the studies reported below. We conveniently maintained spheroids in the same 384-microwell plate used for spheroid formation by robotic exchange of culture medium.

### Effect of TNBC-stromal cells signaling on cellular metabolic activity of spheroids

**Figure 2:**
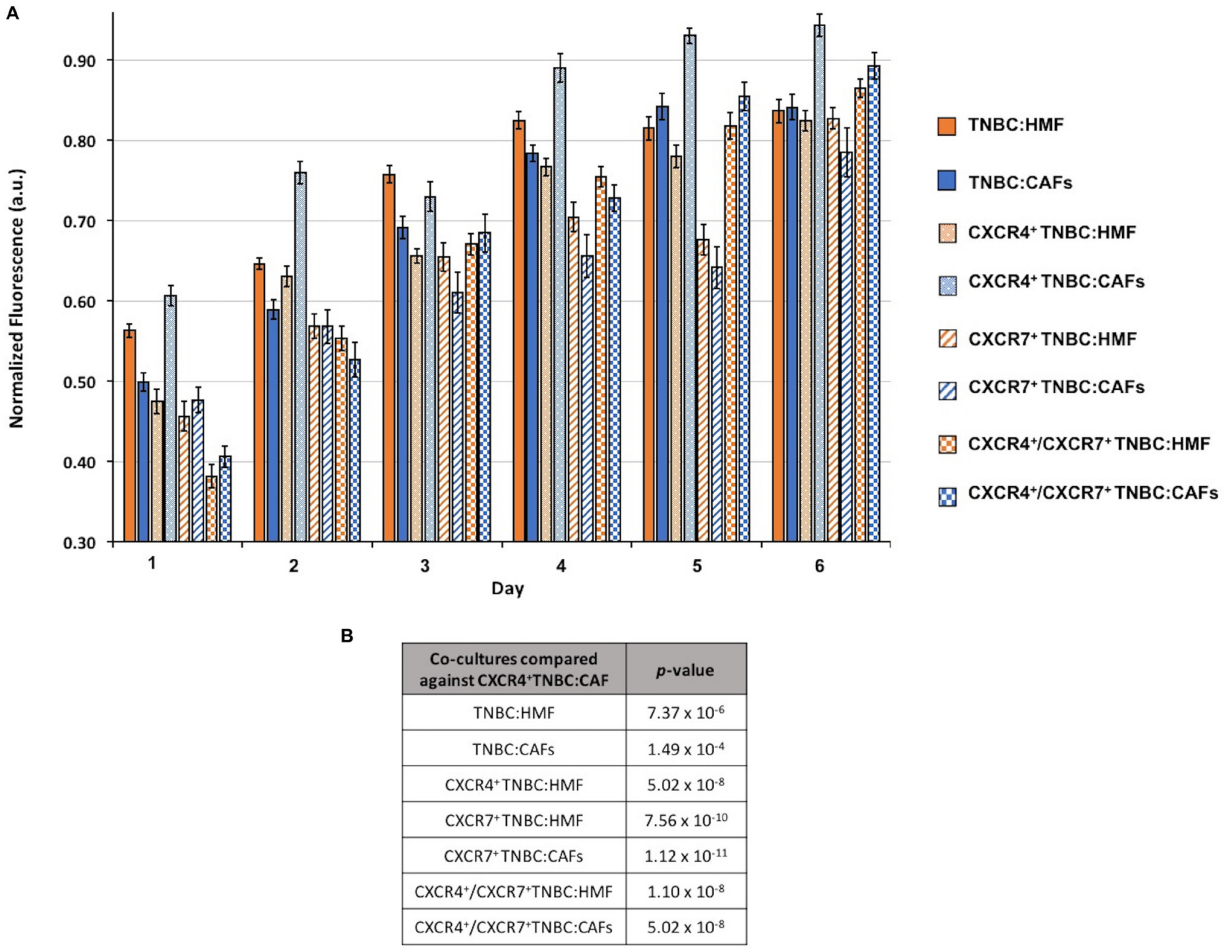
(**A**) Growth of co-culture spheroids based on metabolic activity measurements is shown over a six-day period. Spheroids consist of 1.5 × 10^4^ cells at a ratio of 1:2 TNBC:fibroblasts at the starting point of experiments. (**B**) *p*-values from a statistical test show that the CXCR4^+^TNBC:CAFs co-culture model is consistently and significantly more proliferative than all the other models.

To investigate the effect of CXCL12 signaling through CXCR4 and/or CXCR7 receptors on the proliferation of cells in 3D cultures, we generated eight different co-culture spheroids that contained at least one element of this chemokine-receptor(s) signaling or completely lacked them: (i) TNBC:HMF, (ii) TNBC:CAFs, (iii) CXCR4^+^TNBC:HMF, (iv) CXCR4^+^TNBC:CAFs, (v) CXCR7^+^TNBC:HMF, (vi) CXCR7^+^TNBC:CAFs, (vii) CXCR4^+^/CXCR7^+^TNBC:HMF, and (viii) CXCR4^+^/CXCR7^+^TNBC:CAFs. The co-culture spheroids of TNBC:HMF that lacked this signaling served as the global negative control. Spheroids were evaluated for their cellular proliferation over a six-day period following formation and in medium containing only 1% FBS at the starting point and no renewal (Figure 2A). The TNBC:CAFs model displayed either a lower or fairly similar activity compared to the TNBC:HMF model, indicating that CXCL12 production by CAFs does not confer growth advantage to TNBC cells lacking the cognate receptors of the chemokine. On the other hand, the CXCR4^+^TNBC:CAFs model showed overall the highest and continuously increasing activity throughout the culture compared to all the other co-culture spheroids. The CXCR4^+^TNBC:CAFs model also consistently showed 11–28% greater activity than its CXCR4^+^TNBC:HMF counterpart lacking the CXCL12 chemokine as determined by a linear regression analysis. There was no significant difference between cell proliferation in CXCR7^+^TNBC:HMF and CXCR7^+^TNBC:CAFs co-culture spheroids. Interestingly, these models even had lower activity level than the TNBC:HMF and TNBC:CAFs spheroids on each day of measurement. When the effect of expression of both CXCR4 and CXCR7 receptors on cell proliferation was evaluated, the CXCR4^+^/CXCR7^+^TNBC:CAFs model had slightly increased activity, up to 4.5%, than the CXCR4^+^/CXCR7^+^TNBC:HMF spheroids on certain days. Our statistical analysis of the multivariate, temporally-dependent data resulting from all eight co-culture spheroid models showed that cell proliferation of the CXCR4^+^TNBC:CAFs model was significantly different from the seven other models across the six-day culture period (Figure 2B).

Consistent with reports in mouse models [49–51], our finding suggests that the CXCR4 – CXLC12 axis is a key mediator of cell proliferation in breast cancer. The effect of CXCR4 – CXLC12 signaling on promoting primary and metastatic breast tumor growth has been demonstrated in animal models and clinical studies [52, 53]. Several studies have reported that CXCR7 is a scavenger receptor to prevent saturation of CXCR4 receptors and also to create CXCL12 gradients to facilitate migration of CXCR4^+^ cancer cells [17, 54–57]. This provides a plausible explanation for the similar activity levels of the CXCR7^+^TNBC:HMF and CXCR7^+^TNBC:CAFs co-culture models. We note that the role of the CXCR7 receptor in the presence of CXCL12 signaling remains disputed [58–60]. For example, studies show that CXCR7 expression lead to growth of primary breast tumors in rat models through promoting angiogenesis, which is consistent with data showing upregulation of this receptor on tumor vasculature [61–63]. However, results from these studies cannot be correlated with those from our 3D cultures that represent models of avascular tumors. Altogether, these data demonstrate that our 3D cultures recapitulate growth properties of breast tumors. Based on analysis of the data, we selected the CXCR4^+^TNBC:CAFs model and its respective negative control, CXCR4^+^TNBC:HMF, for mechanistic studies of CXCR4 – CXLC12 signaling in TNBC.

### Inhibition and stimulation of CXCL12 – CXCR4 signaling

**Figure 3:**
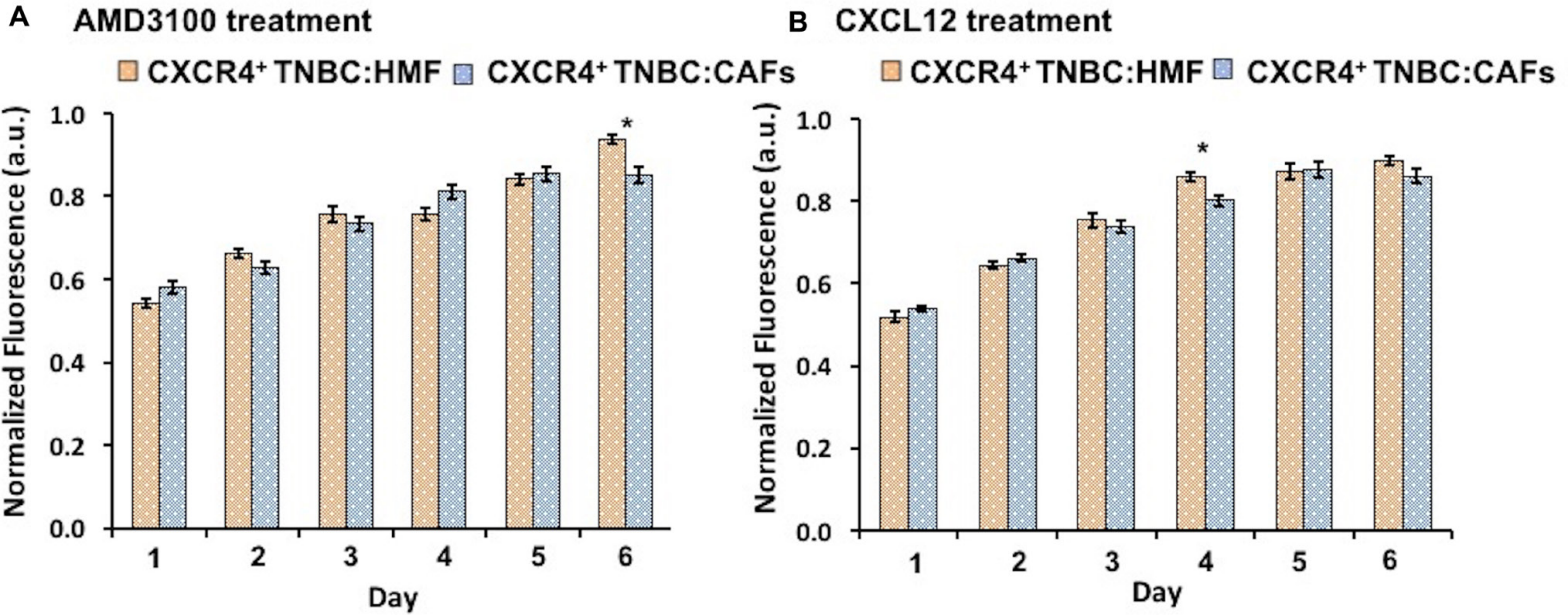
(**A**) Treatment of CXCR4^+^TNBC:CAFs co-culture spheroids with AMD3100 (CXCR4 antagonist) normalizes proliferation to level of the CXCR4^+^TNBC:HMF co-culture. (**B**) Treating CXCR4^+^TNBC:HMF co-culture spheroids with CXCL12-containing conditioned medium from CAFs stimulates proliferation to that of CXCR4^+^TNBC:CAF co-culture spheroids.

We conducted two sets of experiments to evaluate if the higher proliferative activity of the CXCR4^+^TNBC:CAFs model was indeed due to CXCR4 – CXLC12 signaling. The experiments included an inhibition test and a stimulation test. First, we treated spheroids with AMD3100, an antagonist of CXCL12 – CXCR4 that blocks the CXCR4 receptor [64, 65]. Consistently throughout the six-day culture, this treatment reduced proliferation of the CXCR4^+^TNBC:CAFs model to the level of the CXCR4^+^TNBC:HMF model that lacks CXCL12 (Figure 3A). Next, we treated the CXCR4^+^TNBC:HMF model with CXCL12-containing conditioned medium of CAFs. This stimulation elevated the proliferation of the CXCR4^+^TNBC:HMF model to the level of the CXCR4^+^TNBC:CAFs model (Figure 3B). In both cases, our analysis showed minimal statistically significant differences between the treated and non-treated co-culture models. Collectively, these results established that greater proliferation of the CXCR4^+^TNBC:CAFs spheroid model than the CXCR4^+^TNBC:HMF spheroid model was due to CXCR4 – CXCL12 signaling.

### Evaluation of cancer cell growth in spheroids due to CXCL12 – CXCR4 signaling

**Figure 4:**
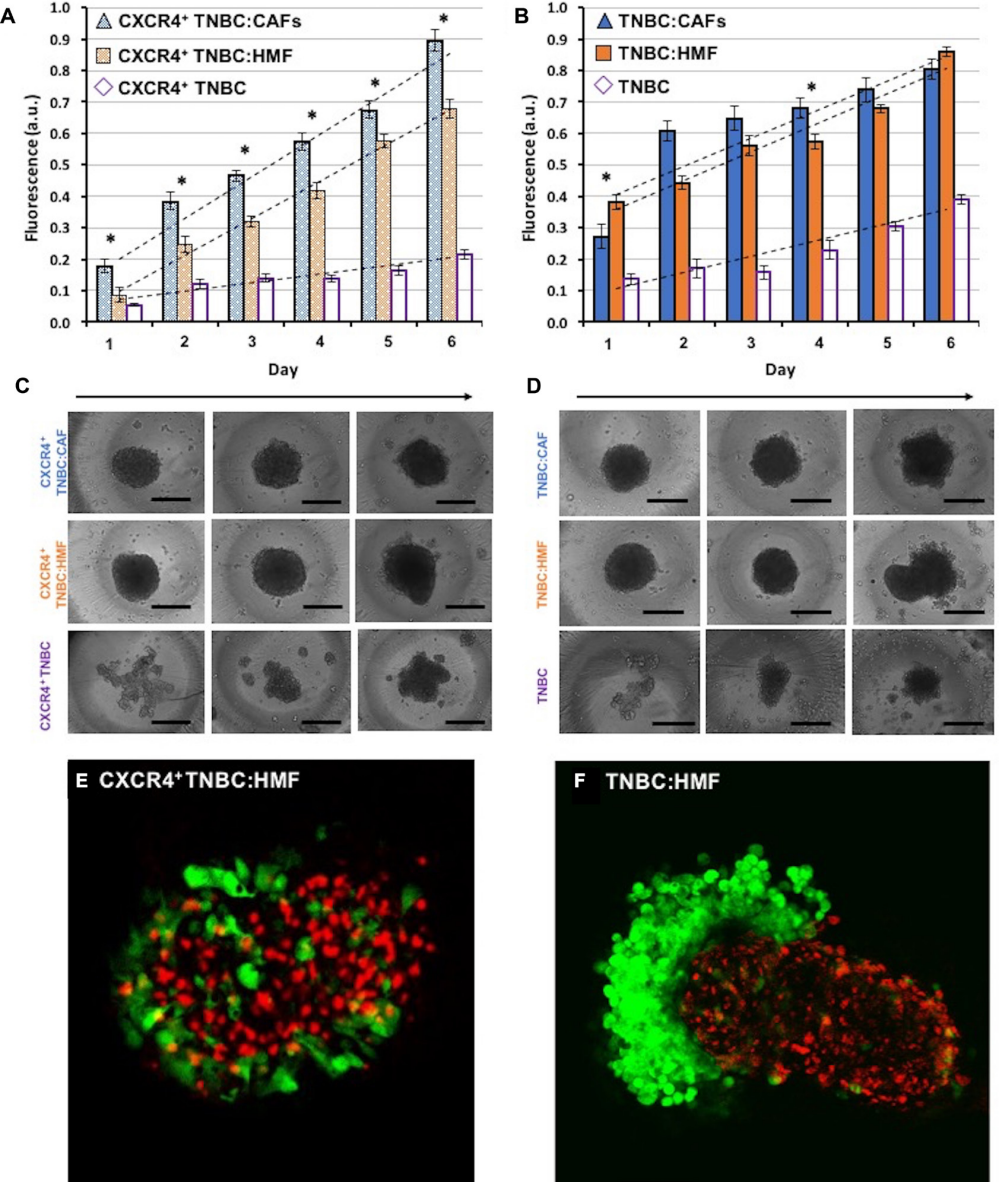
Proliferation of TNBC cells in co-culture spheroids measured using their endogenous eGFP signal (**A**) TNBC cells in the CXCR4^+^TNBC:CAFs model display consistently greater proliferation compared to spheroids of CXCR4^+^TNBC:HMF co-culture model and CXCR4^+^TNBC single culture with a larger slope of 11.4% and 77.1%, respectively. (**B**) TNBC cells lacking CXCR4 expression in co-culture with HMF and CAFs show a similar proliferation. (**C–D**) Morphology of resulting co-culture spheroids and mono-culture spheroids of breast cancer cells on days 1, 3, and 6 (left to right). Error bars represent standard error of mean. (**E–F**) Confocal images of the co-culture spheroids containing HMF cells on day 4. Scale bar is 200 µm. ^*^p < 0.05.

The above study based on metabolic activity of cells provided a measure of stromal-cancer cells signaling effect on the overall growth of spheroids. Considering that activated stromal cells (CAFs) in breast tumors also proliferate, we investigated to what extent greater overall growth results from proliferation of CXCR4^+^ TNBC cells in CXCR4^+^TNBC:CAFs spheroids compared with other spheroid models shown in Figure 2A. Measurements of the endogenous eGFP signal of the cancer cells showed that cancer cells in the CXCR4^+^TNBC:CAFs model display a statistically significant (*p* < 0.05) growth increase throughout the measurement window (Figure 4A). Linear regression analysis showed that CXCR4^+^TNBC cells in this model had an 11.4% larger growth slope over time than their counterparts in the CXCR4^+^TNBC:HMF model. This indicates that CAFs confer increasingly greater proliferative activity to the breast cancer cells. To validate this result, we evaluated growth of cancer cells in co-culture spheroids of TNBC:HMF and TNBC:CAFs that lacked both elements of the signaling axis or CXCR4 receptor expression, respectively. Despite some random differences in growth of cancer cells between the two models on several days, the slope of linear regression was smaller by 2% in the co-culture containing CAFs (Figure 4B). Thus, these results established the role of CXCR4 – CXCL12 signaling on enhanced growth of breast cancer cells [66, 67].

TNBC cells in all four co-culture spheroid models displayed a larger growth than their respective mono-culture spheroids generated with the same density of 5 × 10^3^ breast cancer cells. The slope of linear regression of growth curves was 5.0 folds and 1.8 folds larger for the CXCR4^+^TNBC:CAFs and TNBC:CAFs co-culture models relative to their mono-culture breast cancer cell spheroids, respectively. Similarly, the regression line slope was 4.4 folds and 1.8 folds larger for the CXCR4^+^TNBC:HMF and TNBC:HMF co-culture models relative to their mono-culture breast cancer cell spheroids, respectively. This implies a major role for heterotypic cellular signaling in tumors and suggests that breast cancer cells induce the normal fibroblasts to produce soluble factors to promote growth of malignant cells. Studies show that cancer cells produce growth factors such as transforming growth factor beta (TGF-β), platelet-derived growth factor (PDGF), and fibroblast growth factor 2 (FGF2) that activate the adjacent stromal cells, which in turn secrete signaling molecules such as hepatocyte growth factor (HGF) to promote proliferation of cancer cells [10, 16, 68–71]. The larger growth rate of cancer cells in the CXCR4^+^TNBC:HMF co-culture spheroids compared to the respective CXCR4^+^TNBC mono-culture spheroids may be mediated by soluble factors secreted by CXCR4-expressing cancer cells to transform HMF cells to CAFs [72].

Morphologically, mono-culture spheroids of CXCR4^+^TNBC and TNBC became more compact by day six of culture. Co-culturing breast cancer cells and fibroblasts significantly enhanced compactness of spheroids earlier, indicating that fibroblasts facilitate spheroid formation of breast cancer cells (Figure 4C–4D) [73]. The co-culture models containing CXCR4^+^TNBC cells exhibited a compact morphology for several days with minimal increases of volume (9–15%) from days 1 to 3. But over time, the co-culture spheroids of CXCR4^+^TNBC and HMF cells showed separation of fibroblast and cancer cells with an 82% volume increase from days 3 to 6. This is likely due to the expression of incompatible junctional proteins in breast cancer and fibroblast cells [74]. For example, it was shown that intercellular adhesion was dependent on cadherins expression of each cell type, and only cells that expressed the same type of cadherins intermixed [75]. Notably, the separation behavior was observed considerably more in the TNBC:HMF co-culture model and was further demonstrated through confocal imaging in Figure 4E–4F. Expression of CXCR4 on TNBC cells largely prevented separation from HMF cells in the CXCR4^+^TNBC:HMF model. Additionally, inclusion of CAFs, regardless of CXCR4 expression on TNBC cells, blocked the segregation effect and helped the spheroids retain a compact morphology, mimicking the unique characteristic of CAFs to infiltrate tumor masses [76]. All spheroids displayed shedding of TNBC cells from their peripheries on later days of culture, suggesting that active proliferation of border cells is somewhat compensated by cell shedding [77]. We note that a thorough understanding of the underlying mechanisms of these morphological differences in co-culture spheroids requires a more extensive analysis.

### Molecular analysis of CXCL12 – CXCR4 signaling on cancer cell proliferation in spheroids

**Figure 5:**
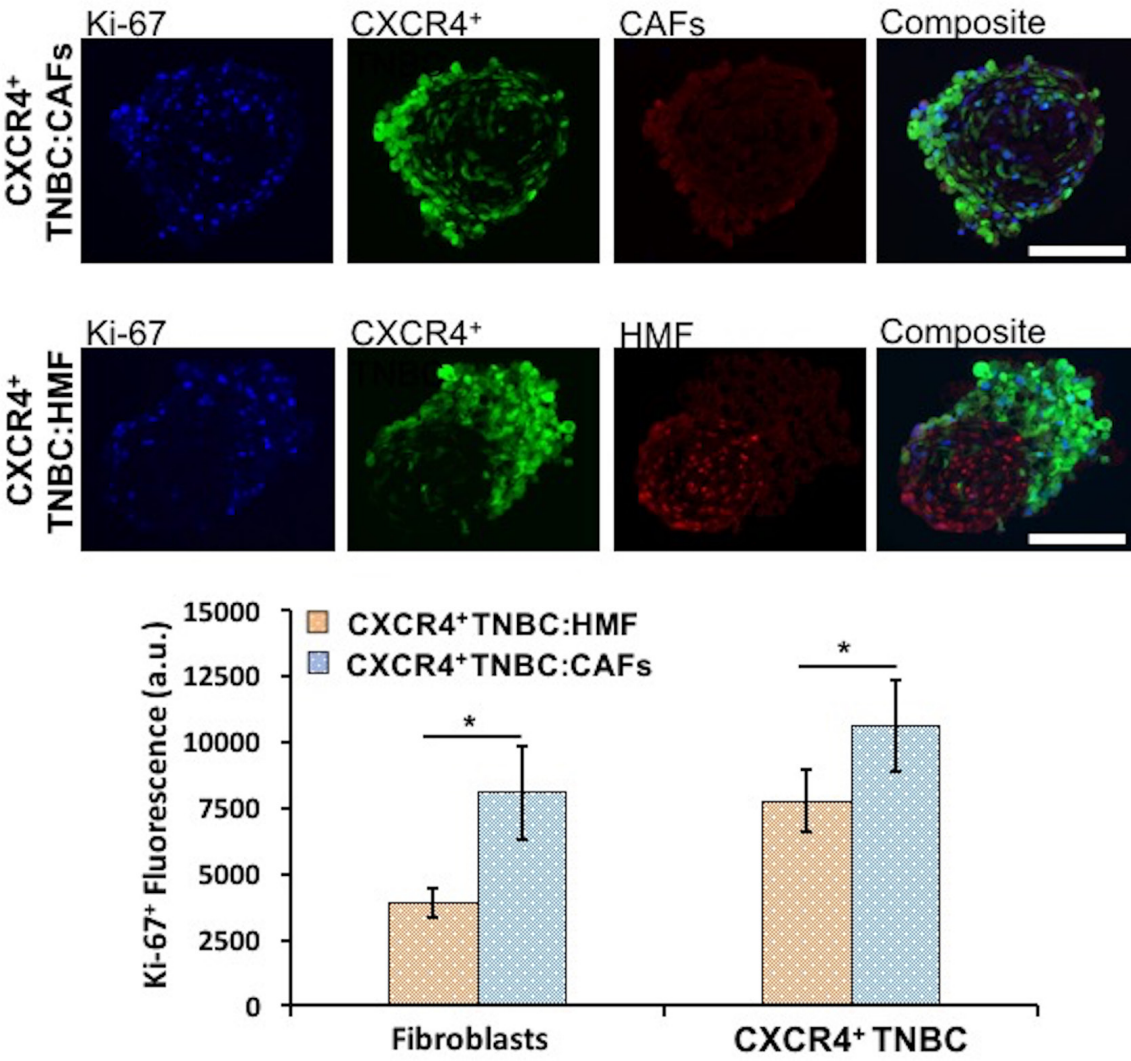
Proliferation of CXCR4^+^TNBC cells and fibroblasts (HMF and CAFs) in co-culture spheroids characterized through measurements of Ki-67^+^ proliferative cells using immunostained images of cryosections of 4-day old spheroids. Both cancer cells and fibroblasts show significantly greater proliferation in the CXCR4^+^TNBC:CAFs co-culture model (blue bars). Scale bar is 200 µm. ^*^p < 0.05.

We evaluated proliferation of both breast cancer and stromal cells in the CXCR4^+^TNBC:CAFs and CXCR4^+^TNBC:HMF co-culture spheroids using IHC analysis of cryosections of spheroids and quantifying Ki-67^+^ proliferative cells. Breast cancer and stromal cells showed statistically significant higher proliferation (*p* < 0.05) in the CXCR4^+^TNBC:CAFs model (Figure 5). When co-cultured with CAFs, the CXCR4^+^TNBC cells showed 1.4 times greater Ki-67 staining, in agreement with results based on eGFP signal measurements (Figure 4). Additionally, CAFs in the co-culture model had a remarkably two-fold higher Ki-67 staining compared to HMF cells. This is consistent with increased proliferation of CAFs in tumor tissues [10, 78], and the overall greater stromal content in more aggressive and larger tumors [6, 79, 80]. We note that the CAFs secrete mCherry-tagged CXCL12 into the medium, whereas the HMF cells contain mCherry signal in their nuclei. This is the reason for visual differences between the red fluorescence images of CAFs and HMF cells in Figure 5. To avoid relying on the mCherry signal for quantification of cell proliferation, we used the Ki-67^+^ and eGFP signal of breast cancer cells to quantify proliferative TNBC cells, and then subtracted this value from the total Ki-67^+^ fluorescence to quantify proliferation of stromal cells (CAFs or HMF) in their respective co-cultures.

**Figure 6:**
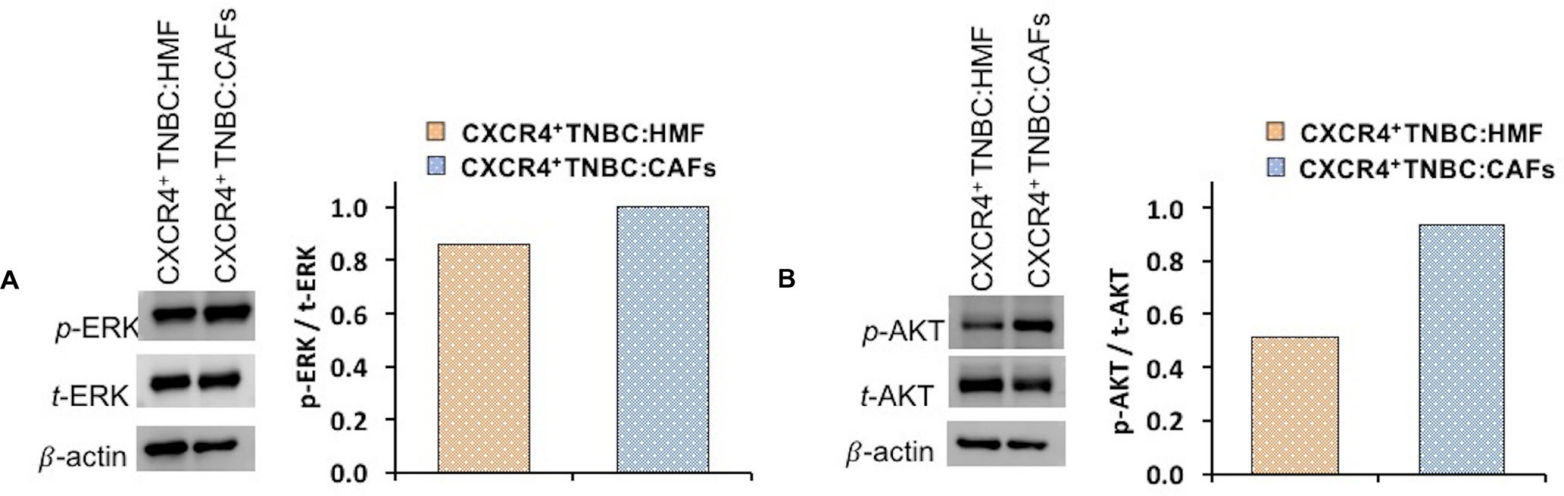
Western blot analysis of signaling proteins (**A**) *p*-ERK and (**B**) *p*-AKT in co-cultures of CXCR4^+^TNBC cells with HMF and CAFs. Quantified data were normalized to β-actin protein expression.

Next, we investigated molecular mechanisms for increased proliferation of stromal and breast cancer cells in the CXCR4^+^TNBC:CAFs spheroid model. The CXCL12 – CXCR4 axis may activate multiple pathways in cancer cells [17, 81–83]. We focused on ERK and AKT, two prominent drivers of proliferation in breast cancer and other malignancies [84]. Western blot analysis relative to the total protein and β-actin expression of each co-culture system showed that cells in CXCR4^+^TNBC:CAFs spheroids had moderately higher levels of *p*-ERK (Figure 6A) and substantially higher levels of *p*-AKT (Figure 6B) compared to CXCR4^+^TNBC:HMF spheroids. The displayed immunoblots were quantified (normalized to their respective total and β-actin protein expressions), showing an 81% increase in the *p*-AKT/*t*-AKT levels and a 16% increase in *p*-ERK/*t*-ERK levels in the CXCR4^+^TNBC:CAFs spheroids. Although *p*-ERK increase is modest, small changes in activation of MAPK components produce much larger effects on overall signaling and biologic outputs [85]. These results suggest that the CXCL12 – CXCR4 signaling upregulates activation of the PI3K and MAPK pathways to support TNBC cells metabolic and growth activities.

**Figure 7:**
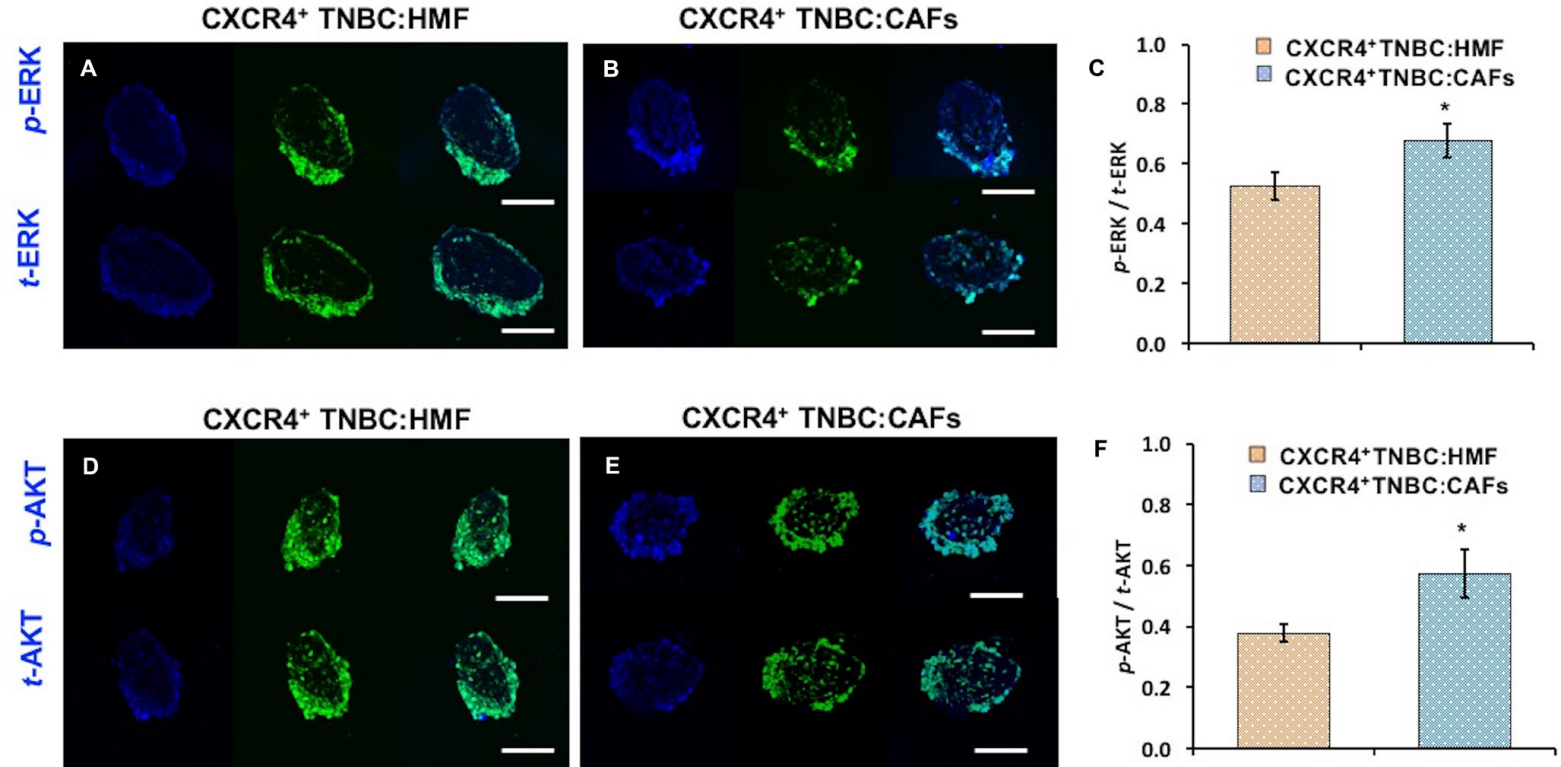
Cryosections of co-culture spheroids of CXCR4^+^TNBC cells (green) with HMF and CAFs immunostained for (**A–B**) ERK and (**D–E**) AKT. A blue fluorescent secondary antibody was used to detect ERK and AKT. Images represent spheroids on day 4 of culture. Quantification showed higher (C) ERK and (**F**) AKT phosphorylation in the CXCR4^+^TNBC cells when co-cultured with CAFs. Scale bar is 200 µm. ^*^p < 0.05.

To further substantiate these results, we conducted IHC analysis of cryosections of co-culture spheroids for ERK and AKT phosphorylation (Figure 7A–7B, 7D–7E). Quantification of immunostained sections (normalized to total protein expression and highest fluorescent value obtained) showed a similar pattern to Western blotting with a 52% and 28% increase in *p*-AKT/*t*-AKT and *p*-ERK/*t*-ERK levels, respectively, in the CXCR4^+^TNBC:CAFs model (Figure 7C, 7F). We note that high levels of ERK and AKT at the periphery of immunostained sections is due to the positioning of CXCR4^+^TNBC cells at the periphery of the co-culture spheroids. Fibroblasts were mainly distributed in the center of the sections and showed minimal staining, clear from the composite image in Supplementary Figure 2 and Supplementary Movie 1. Therefore, quantification was only performed on the CXCR4^+^TNBC cells of stained sections.

### Drug sensitivity of stromal-cancer cells co-culture spheroids

**Figure 8:**
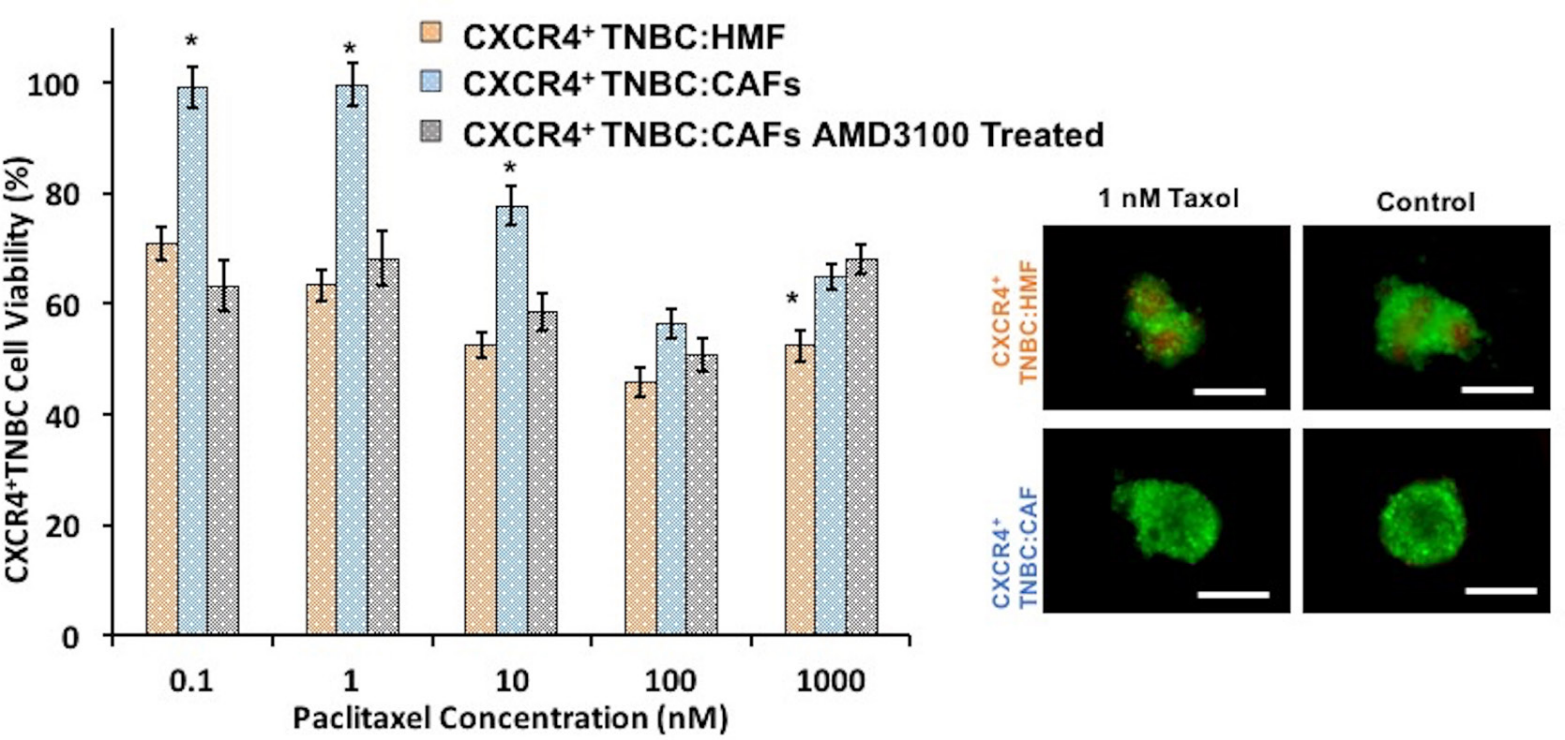
CXCR4^+^TNBC:CAFs co-culture spheroids (blue bars) display resistance to paclitaxel treatment but lose resistance to the drug when co-treated with a CXCR4 receptor antagonist AMD3100 (gray bars), similar to the negative control counterpart (CXCR4^+^TNBC:HMF, orange bars). Images reflect spheroids at the end of drug treatment period (6 days). Scale bar is 200 µm. ^*^p < 0.01.

Biochemical interactions of activated stromal cells and cancer cells are suggested as a major cause of drug resistance. To demonstrate the effect of CXCL12 – CXCR4 signaling on drug response of breast cancer cells, we evaluated the sensitivity of co-culture spheroids to a standard chemotherapy drug, paclitaxel, to which TNBC patients may develop resistance [86–88]. Cancer cells in CXCR4^+^TNBC:CAFs co-culture spheroids were significantly more resistant to paclitaxel at a 0.1–10 nM concentration range than cancer cells in the CXCR4^+^TNBC:HMF spheroids (*p* < 0.01). The largest difference in cancer cell viability (36%) occurred at a drug concentration of 1 nM (Figure 8). This demonstrates the impact of activated tumor stromal cells on chemotherapy resistance of cancer cells, consistent with several other reports [89–92], and that our 3D tumor models recapitulate stromal-mediated drug resistance. Treatment with the CXCR4 antagonist AMD3100 eliminated resistance to paclitaxel of cancer cells in the CXCR4^+^TNBC:CAFs model. This result emphasizes the potential to improve response to chemotherapy by targeting the CXCL12-CXCR4 signaling [93–95].

**Figure 9:**
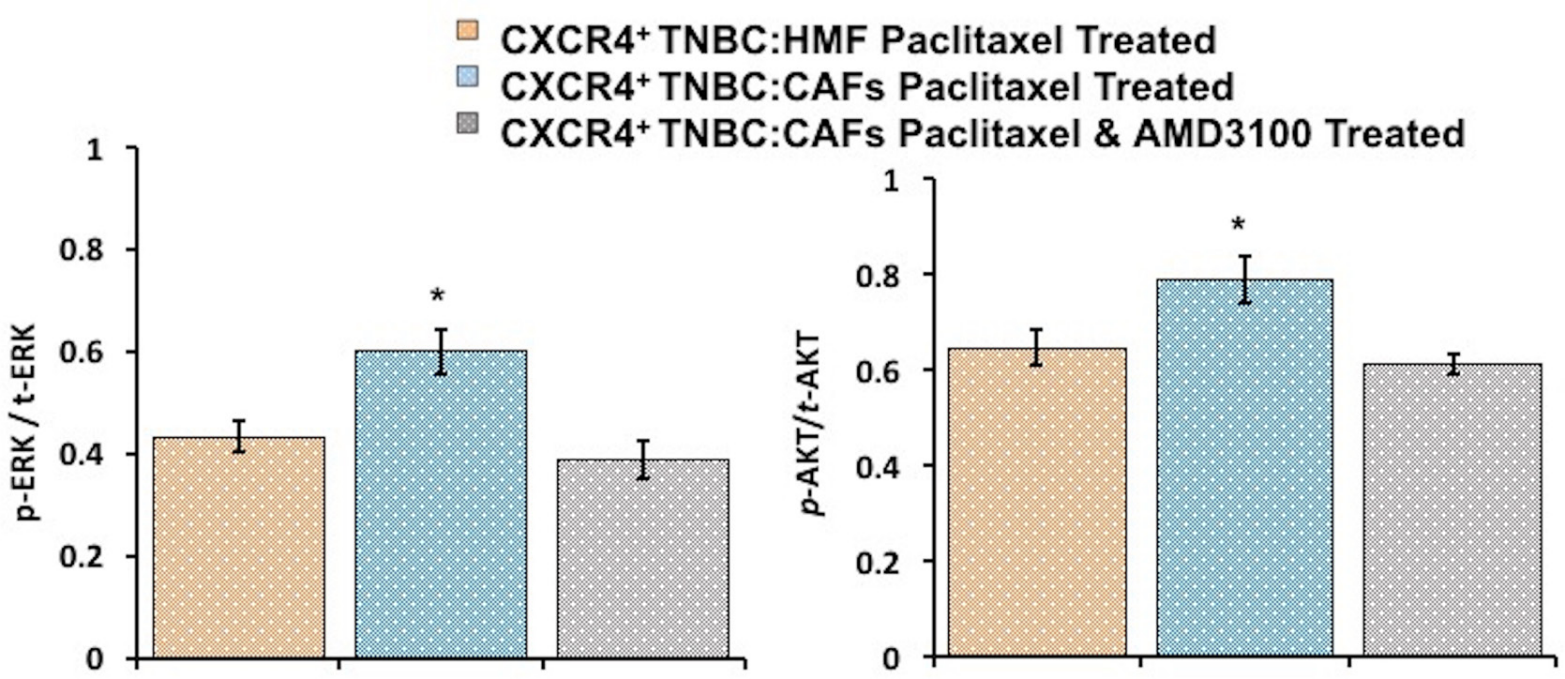
Immunohistochemical analysis of drug-treated spheroids indicates that increases in AKT and ERK phosphorylation levels in CXCR4^+^TNBC:CAF co-culture spheroids contribute to paclitaxel resistance (blue bars). Co-treatment with a CXCR4 receptor antagonist, AMD3100, lowers AKT and ERK activities (gray bars) to that observed in the paclitaxel sensitive control culture (CXCR4^+^TNBC:HMF, orange bars). ^*^p < 0.05.

To understand the mechanism of paclitaxel resistance, we performed IHC analysis of cryosections of spheroids to assess ERK and AKT activity in CXCR4^+^TNBC:CAFs and CXCR4^+^TNBC:HMF models treated with 1 nM paclitaxel. Following treatment, significantly higher phosphorylation levels of both proteins were maintained in the CXCR4^+^TNBC:CAFs model (*p* < 0.05), indicating that resistance to paclitaxel due to the CXCL12 – CXCR4 signaling is mediated by activation of these kinase pathways (Figure 9 and Supplementary Figure 3). Previous studies showed that paclitaxel resistance strongly correlated with increased activation of the MAPK and PI3K pathways in breast cancers [96–99], supporting our data. Upon co-treatment of spheroids with 1 μM of AMD3100, phosphorylated levels of ERK and AKT in the CXCR4^+^TNBC:CAFs model treated with paclitaxel normalized to that of control CXCR4^+^TNBC:HMF spheroids. These results collectively demonstrate the promising therapeutic approach of sensitizing TNBC tumors to chemotherapy treatment by disrupting the cancer-stroma signaling and its downstream pathways to overcome stroma-induced cancer cell growth and chemotherapy resistance [100, 101].

**Figure 10:**
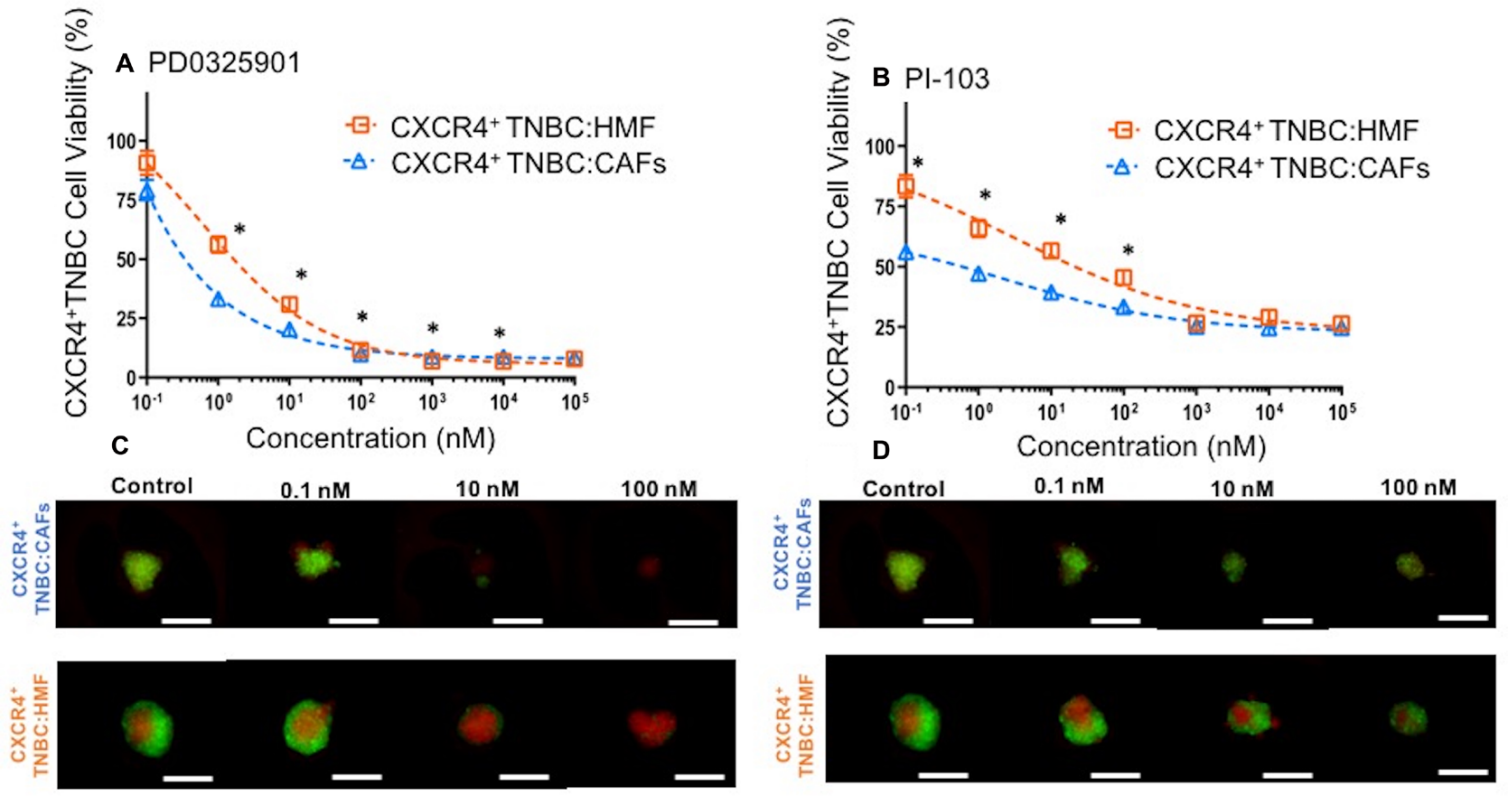
Viability of CXCR4^+^TNBC cells in co-culture with HMF and CAFs treated with a MEK inhibitor, (**A**) PD0325901, and a PI3K inhibitor, (**B**) PI-103, measured using the endogenous eGFP signal of the TNBC cells. CXCR4^+^TNBC cells in co-culture with CAFs show greater drug sensitivity to the molecular inhibitors. Images reflect spheroids at end of drug treatment period (5 days). Error bars represent standard error of mean. Scale bar is 200 µm. ^*^p < 0.05.

Our findings suggest that greater proliferation and drug resistance of cancer cells in the CXCR4^+^TNBC:CAFs model is due to CXCL12 – CXCR4 signaling through AKT and ERK. Thus, we hypothesized that inhibition of these kinase pathways may block enhanced growth of cancer cells and compromise their viability. We treated both co-culture spheroids with specific inhibitors of MEK/ERK (PD0325901) and PI3K/AKT (PI-103) at a wide concentration range for a six-day period. Effectiveness of the inhibitors was assessed by eGFP detection of breast cancer cells in treated groups relative to their respective negative control group (no treatment) and presented as percent cell viability. We found that breast cancer cells in the CXCR4^+^TNBC:CAFs model were more sensitive to PD0325901 treatment below 100 nM (Figure 10A) and PI-103 treatment below 1 µM (Figure 10B). We further confirmed the reduction in breast cancer cell viability using quantification of fluorescence images of drug-treated spheroids in Figure 10C–10D. The largest differences in the viability of CXCR4^+^TNBC cells in co-culture with CAFs and HMF were 23% and 27% at 1 nM of PD0325901 and 0.1 nM of PI-103, respectively. Interestingly, PI-103 at a 0.1 nM concentration reduced breast cancer cell viability in the CXCR4^+^TNBC:CAFs model to 56%. This was consistent with protein expression results that showed significant activation of the PI3K/AKT pathway (Figure 6 and 7) leads to increased growth of the breast cancer cells (Figure 4). This also agrees with a study that showed CXCL12 signaling-mediated drug resistant cancer cells became substantially more sensitive to chemotherapy through AKT inhibition [90]. At higher concentrations, the MEK/ERK pathway inhibitor is more effective and reduces the cancer cells viability to less than 10%. Overall, the results corroborate our protein expression study that showed upregulated activity of these two kinase pathways due to CXCL12 – CXCR4 signaling. Although CXCL12 – CXCR4 can potentially activate multiple pathways in cancer cells [102], these results establish signaling to MAPK and PI3K pathways in TNBC cells and the promising result of targeting these pathways to improve cytotoxicity. Considering the activation of both kinase pathways due to this signaling, their simultaneous inhibition using combination treatments with specific molecular inhibitors of AKT and MAPK pathways may result in synergistic inhibition of TNBC cell growth and survival at lower drug concentrations than those with single-agent treatments [103]. Collectively, these results strongly support the utility of our 3D tumor models for cellular and molecular studies of tumor-stromal interactions.

In conclusion, our robotic technology allowed facile generation of co-culture spheroids to conveniently study stromal-cancer (TNBC) cells interactions. We found that CXCL12 from CAFs signaled through CXCR4 on triple negative cancer cells to significantly enhance TNBC cell proliferation. Additionally, CAFs showed greater proliferation than their normal HMF counterpart. Increased proliferation of TNBC cells occurred due to activation of MAPK and PI3K signaling pathways, two known effectors of CXCR4 signaling. CXCL12 – CXCR4 signaling also conferred resistance to chemotherapy. We demonstrated the potential to overcome stromal-mediated drug resistance using small molecule inhibitors of MAPK and PI3K pathways and CXCR4 signaling. Altogether, these findings highlight the power of our model system to identify promising new therapeutic strategies to improve response to therapy in TNBC. Future studies with improved tumor models should address and distinguish effects of stromal cells-deposited ECM signaling and stromal cells-mediated biochemical signaling with cancer cells on the proliferation and drug response of cancer cells.

## MATERIALS AND METHODS

### Aqueous two-phase system (ATPS) preparation

Two polymers, BioUltra polyethylene glycol (PEG) (Sigma), MW: 35 kDa, and dextran (DEX) (Pharmacosmos), MW: 500 kDa, were utilized to form an aqueous two-phase system, as described previously [38, 104]. Briefly, PEG and DEX were added to the complete cell culture medium at 5.0% (w/v) and 12.8% (w/v), respectively. The polymers were dissolved using a vortex mixer and then incubating in a water bath at 37°C for 1 hour. The polymer solutions were then stored at 4°C until use. Prior to use, the PEG phase polymer solution was filtered by passing it through a 0.2 μm syringe filter to remove any impurities.

### Cell culture

Three different triple negative breast cancer cells were utilized for experiments. The cell lines were: MDA-MB-231 (labeled TNBC), MDA-MB-231 cells expressing the CXCR4 receptor (labeled CXCR4^+^TNBC), and MDA-MB-231 cells expressing the CXCR7 receptor (labeled CXCR7^+^TNBC). These cells were previously stably transduced using lentiviral vectors and the expression of CXCR4 and CXCR7 receptors on cancer cells was confirmed through flow cytometry [105]. The TNBC cells were additionally stably transduced to express eGFP for detection [105]. In addition, a human mammary fibroblast (HMF) cell line (labeled HMF) stably transduced with mCherry protein was utilized [106]. HMF cells were also stably transduced to secrete 0.75 ng/ml/hr of CXCL12-α fused with mCherry for tracking, and the resulting cells were labeled as CAFs [56, 107]. CAFs extracted from breast cancer patients secreted varying amounts of CXCL12 in media ranging from 0.9 ng/ml – 2 ng/ml over a 48 hr time period, depending on the patients and the tumor samples [16]. The higher CXCL12 secretion rate of our transduced HMFs accounts for medium renewals and drug additions that consequently dilute the CXCL12 concentration in the medium. To maintain the cells in culture, they were grown in T175 flasks at 37°C and 5% CO_2_ using Dulbecco’s Modified Medium (DMEM) (Sigma) supplemented with 10% fetal bovine serum (FBS) (Sigma), 1% glutamine (Life Technologies), and 1% antibiotic (Life Technologies). Once a confluent monolayer formed, cells were rinsed with phosphate buffered saline (PBS) (Sigma) and dissociated using 5 ml trypsin (Life Technologies) in an incubator for ∼2 min. The cells were then neutralized with 10 ml of the complete growth medium, collected, and centrifuged for 5 min at 1000 rpm. The supernatant was aspirated, cells were resupsended in 1 ml of fresh medium, and loaded into a hemocytometer for counting.

### Co-culture spheroid formation using aqueous two-phase system (ATPS)

A ratio of 1:2 TNBC to stromal cells was used to form co-culture spheroids that model the high stromal content often observed in larger and more advanced human breast tumors [5–8]. Our initial tests with 1:3 and 1:4 ratios (TNBC cells to fibroblasts) while keeping the initial TNBC cell density constant did not make any further changes in the growth of cancer cells. Therefore, the selected ratio of 1:2 (TNBC cells to fibroblasts) was used for all the studies. To prepare for forming spheroids, 30 μl of the PEG phase solution was dispensed into each well of a 384-well plate, labeled as the destination plate. The DEX phase solution was mixed at an equal volume with a specific density of cells (1 × 10^4^/0.3 μl for monoculture spheroids of TNBC cells, 2 × 10^4^/0.3 μl for monocultures spheroids of HMF and CAFs, and 3 × 10^4^/0.3 μl for co-culture spheroids of TNBC-HMF and TNBC-CAFs). The DEX phase solution and the cell suspension became diluted in half upon mixing resulting in a final polymer concentration of 6.4% (w/v), and densities of 5 × 10^3^/0.3 μl TNBC cells in monoculture spheroids, 1 × 10^4^/0.3 μl HMF cells or CAFs in monoculture spheroids, and 1.5 × 10^4^/0.3 μl in co-culture spheroids of TNBC-HMF cells or TNBC-CAFs. A source 384-well plate was prepared by loading the wells from a single column with ∼20 μl of the solution of mixed DEX phase and cell suspension. A robotic liquid handler (Bravo SRT, Agilent) was programmed to mix the source plate content and aspirate 0.3 μl from each well. This solution was robotically dispensed as a single drop into each well of the destination plate containing the PEG phase. This process was repeated for all columns of the destination plate. After completion, the destination plate was placed in an incubator to allow cells to aggregate into a single spheroid in each well within 48 hrs. Our studies used a total of eight co-culture models: TNBC:HMF, TNBC:CAFs, CXCR4^+^TNBC: HMF, CXCR4^+^TNBC:CAFs, CXCR7^+^TNBC:HMF, CXCR7^+^TNBC:CAFs, CXCR4^+^/CXCR7^+^TNBC:HMF, and CXCR4^+^/CXCR7^+^TNBC:CAFs. Images of co-culture spheroids were captured using an inverted fluorescent microscope (Axio Observer, Zeiss) equipped with a high-resolution camera (AxioCam MRm, Zeiss).

### Robotic media exchange of co-culture spheroids

After forming spheroids, the cell culture medium in microwells was robotically exchanged to fresh medium containing only ∼1% FBS. This was to ensure minimizing the effect of serum on cell growth and capture the effect of paracrine stromal-cancer cells signaling [108, 109]. The medium exchange was performed by three consecutive robotic dispensing and aspirating of 40 μl of the medium containing 1% FBS. This reduced the FBS concentration in the microwells from that of the complete growth medium to a very low level of ∼1%. Therefore, the co-culture spheroids were maintained in ∼1% FBS-containing medium for the duration of experiments. The medium exchange also diluted the PEG and DEX polymer concentrations and resulted in a single medium phase. The ATPS was not necessary at this point as it was only used for spheroid formation. The liquid handling protocol was optimized to avoid loss of spheroids during the process of medium exchange. Following this step, spheroids remained in 70 μl of homogenous ∼1% FBS-containing medium for the duration of culture and without any further medium exchange.

### Evaluation of metabolic activity of co-culture spheroids

The growth of co-culture spheroids was evaluated for a six-day period for all eight co-culture models using a standard PrestoBlue metabolic activity assay (Invitrogen). The PrestoBlue reagent contains a resazurin compound that is reduced by metabolically active, viable cells to resorufin detectable with standard plate readers at excitation and emission wavelengths of 560 and 590 nm, respectively [110]. We have previously optimized the PrestoBlue assay for use with spheroid cultures [38, 41]. The resulting raw fluorescence data from all eight co-culture spheroids were normalized to the highest fluorescent readout obtained in the experimental data over the six-day period. Data from replicates (*n* = 14) of each co-culture model were then averaged and statistically evaluated to select certain co-culture models for further studies.

### Statistical analysis of growth of co-culture spheroids

Statistical analysis was performed on the metabolic activity of co-culture models using a one-way multivariate analysis of variance (MANOVA). Briefly, the data represented measured signals from eight different co-culture models over six days. For each model on each day, there were 14 experimental replicates that were repeatedly used over the six-day measurement period. Therefore, we selected one-way MANOVA for data analysis to account for the temporal dependency of the daily measurements with each spheroid from a given co-culture model [111, 112]. The data were tested in two steps: (i) to evaluate if a co-culture model was statistically different from the other models. This was done by testing the null hypothesis that none of the co-cultures was different from the other models; and (ii) to identify if the CXCR4^+^TNBC:CAFs model was statistically different from the other seven models (TNBC:HMF, TNBC:CAFs, CXCR4^+^TNBC:HMFs, CXCR7^+^TNBC:HMF, CXCR7^+^TNBC:CAFs, CXCR4^+^/CXCR7^+^TNBC:HMF, and CXCR4^+^/CXCR7^+^TNBC:CAFs). This was done using seven pairwise comparisons to test the null hypothesis that the CXCR4^+^TNBC:CAFs co-culture model was not different from the other models.

The first statistical test showed significant differences among the eight co-culture models. Next, the CXCR4^+^TNBC:CAFs model was selected for pairwise comparisons with the other seven models due to its higher levels of proliferation. We determined if greater proliferation in this model was statistically significant from the other co-cultures. The Bonferroni correction was utilized to control the family-wise error since this was a multiple testing problem [113]. A significance level of α = 0.05 was selected to determine significant differences between the CXCR4^+^TNBC:CAFs model and every other co-culture model. But α was first modified to α/*m*, where *m* accounts for the number of hypotheses made. In this analysis, the number of hypotheses was *m* = 7. Therefore, a corrected significance level of 0.007 was used to reject the hypothesis that the CXCR4^+^TNBC:CAFs model was not different from all the other seven co-cultures.

For experiments that contained only two or three co-culture models presented in other sections, a student’s *t*-test in Microsoft Excel Software or two-way ANOVA in Minitab Software was utilized with a *p* < 0.05 representing statistical significance.

### Blocking and stimulating chemokine-receptor signaling in co-culture spheroids

To further evaluate cell proliferation due to chemokine-receptor signaling in the CXCR4^+^TNBC:CAFs co-culture spheroids, AMD3100 (Selleckchem) was used to block the signaling and CXCL12-α conditioned medium was used to stimulate the signaling. A stock solution of AMD3100 was prepared in distilled, sterile water at 5 mM and stored at –80°C according to the manufacturer’s instructions. The desired concentration of AMD3100 was prepared by serially diluting the stock solution in 1% FBS-containing cell culture medium. After formation of spheroids in the PEG-DEX ATPS, the medium exchange was done by adding 40 μl of AMD3100 at a 1.75 μM concentration to wells already containing 30 μl of medium. This diluted AMD3100 concentration to 1 μM. The AMD3100 solution was renewed on day four by removing 50 μl of the well contents and adding 50 μl of fresh solution of AMD3100 at a 1 μM concentration.

To induce signaling in the CXCR4^+^TNBC:HMF co-culture spheroids, the spheroids were treated with CXCL12-containing conditioned medium collected from a confluent monolayer of CAFs. The conditioned medium was extracted from the CAFs monolayer 24 hours after seeding to ensure that it contained high levels of nutrients and growth factors. The conditioned medium was added to co-culture spheroids through medium exchange and renewed on day four by removing 50 μl of the well contents and adding 50 μl of fresh conditioned medium. Metabolic activity of co-culture spheroids following inhibition (AMD3100 treatment) or stimulation (CXCL12-α conditioned medium treatment) was evaluated daily using PrestoBlue over the six-day culture period. Data were compared to the respective negative controls, i.e., co-culture spheroids (CXCR4^+^TNBC:HMF for inhibition and CXCR4^+^TNBC:CAFs for stimulation) that were grown in 70 μl of 1% FBS-containing media without receiving any treatment. Daily measured raw data from the co-culture models over the six-day culture were normalized to the highest fluorescent value obtained and then used for statistical tests.

### TNBC cell growth in co-culture spheroids

The growth of TNBC cells in the co-culture models (TNBC:HMF, TNBC:CAFs, CXCR4^+^TNBC:HMF, CXCR4^+^TNBC:CAFs) that were selected based on the metabolic activity analysis was further confirmed by measuring the endogenous eGFP in TNBC cells using a plate reader at excitation and emission wavelengths of 485 and 530 nm, respectively. The results were compared to those of the monoculture spheroids (TNBC and CXCR4^+^TNBC). Each model had *n* = 14 replicates. Data from each model were normalized to an arbitrary fluorescent value of 8000 a.u. for ease of comparison among different groups. Background fluorescence from the mCherry-expressing stromal cells in the co-culture spheroids was subtracted from the measured signal of TNBC cells. Fluorescent images were captured using a confocal microscope (Fluoview, FV1000, Olympus).

### Immunostaining and quantification of proliferation of co-culture spheroids

To validate and quantify cell proliferation of co-culture spheroids, immunohistochemical analysis was performed using an established protocol [41, 114]. Co-culture spheroids were aspirated from microwells on day four and collected in 200 μl microcentrifuge tubes. Medium used for transferring spheroids was carefully removed from the microcentrifuge tubes and 100 μl of 4% formaldehyde was added to fix the spheroids for 10 minutes at room temperature. The spheroids were rinsed with 100 μl of PBS three times for 5 minutes to remove the formaldehyde. A 30% (w/v) sucrose solution was then added to remove water from spheroids and prevent crystal formation upon freezing. Once spheroids sank to the bottom of the microcentrifuge tubes, an equal volume (100 μl) of tissue freezing medium (Triangle Biomedical Sciences) was added to each tube. Samples were kept at 4°C overnight. Frozen samples were prepared the following day by flash-freezing spheroids into biopsy-sized cryomolds with dry ice. The spheroids were embedded between two thin layers of tissue freezing medium while avoiding bubble formation. The frozen molds containing the spheroids were stored at −80°C until use.

A cryostat (Leica CM 1850) was used to cryosection the spheroids to 10 μm-thick slices. Only the middle sections of spheroids were collected and assessed. Each slice was transferred onto Superfrost Plus microscopic slides (Fisher). The sections were stained using a standard immunostaining protocol with primary antibodies purchased from Cell Signaling including the proliferative cell marker Ki-67, phospho-Erk1/2 (phospho-p44/42 MAPK), total-Erk1/2 (p44/42 MAPK), phospho-Akt (Ser473), and total-Akt (pan). A blue fluorescent secondary antibody (AMCA-conjugated goat anti-rabbit, Jackson ImmunoResearch) was used for detection to avoid interfering with the endogenous mCherry and eGFP signals in the stromal and TNBC cells, respectively. The endogenous eGFP fluorescence of TNBC cells was used to distinguish TNBC and stromal cells. An inverted fluorescent microscope (Axio Observer, Zeiss) was used to capture fluorescent images. Image processing and analysis were performed in ImageJ Software (NIH) to quantify expression of each blue-labeled protein. The TNBC cells of sections were found through binary conversion of the green channel images to select and outline only the eGFP-expressing TNBC cells. This TNBC cell outline was then overlaid on the blue channel images to measure the mean gray value of protein-expressing TNBC cells. For Ki-67 analysis, this value was then subtracted from the total mean gray value of blue channel images to determine the mean gray value of Ki-67^+^ stromal cells.

### Western blot analysis with spheroids

Co-culture spheroids (CXCR4^+^TNBC:CAFs and CXCR4^+^TNBC:HMF) were harvested from microplates on day four and transferred into 50 mL conical tubes. After centrifuging down the samples, the supernatant was removed and spheroids were washed with PBS. Spheroids were lysed using 500 μl of complete RIPA buffer (50 mM tric-HCL, 150 mM NaCl 1% NP-40, 0.5% sodium deoxycholate, and 0.1% SDS, pH 7.4 ± 0.2), a protease inhibitor (complete mini, Roche Diagnostics), and phosphatase inhibitor (Life Technologies). Spheroids were sonicated (Vibra-Cell, Sonics) twice for five seconds at a 50% amplitude level to ensure complete lysis. A BCA quantification assay kit (Life Technologies) was then used to quantify the total protein concentration extracted from spheroids. Electrophoresis and electroblotting were performed by protein addition (20 μl) onto a 4–15% gel (Biorad) and then gel transferring onto a nitrocellulose membrane, respectively. The membranes were blocked with 5% BSA (Sigma) for 1 hour. Primary antibodies purchased from Cell Signaling for phospho-Erk1/2 (phospho-p44/42 MAPK), total-Erk1/2 (p44/42 MAPK), phospho-Akt (Ser473), and total-Akt (pan) were prepared at concentrations recommended by the manufacturer, and incubated with membranes overnight at 4°C. The membranes were then incubated with a horseradish peroxidase (HRP)-conjugated secondary antibody for 1 hour. The membranes were thoroughly washed prior to and following secondary antibody treatment and detected with an ECL chemiluminescence detection kit (GE Healthcare) using a FluorChem E imaging system (ProteinSimple). Quantified protein expression data were normalized to their corresponding β-actin expression.

### Chemotherapy drug testing with co-culture spheroids

Both CXCR4^+^TNBC:CAFs and CXCR4^+^TNBC:HMF spheroids were subjected to (i) single treatment with a standard chemotherapy drug, paclitaxel (Selleckchem), and (ii) co-treatment with paclitaxel and AMD3100, to evaluate drug responses of TNBC cells. A stock solution of paclitaxel was prepared in DMSO at a 23 mM concentration and stored at –80°C. For single drug treatment, paclitaxel drug solutions were prepared in 1% FBS-containing cell culture medium at 2X the desired concentration. The drug solutions were added to co-culture spheroids, resulting in a total media volume of 70 μl and diluting the drug concentration in half. For co-treatment of spheroids with paclitaxel and AMD3100, drug solutions were prepared in 1% FBS-containing cell culture medium at 3.5X the desired concentration and directly added (20 μl of each drug solution) to co-culture spheroids in wells already containing 30 μl of media. Paclitaxel was added at a wide range of concentrations (0.01 – 1000 nM) while AMD3100 was used at a constant 1 μM concentration for co-treatments. Single-agent (paclitaxel) and co-treatment (paclitaxel and AMD3100) tests were conducted for six days. A small volume (5 μl) of concentrated AMD3100 (15 μM) was directly added to the co-treated spheroid cultures after 72 hrs of incubation to provide a fresh dose of 1 μM AMD3100. Each condition in drug treatment studies used 14 spheroids. After six days, the TNBC cell viability of drug treated co-culture spheroids was evaluated using their endogenous eGFP fluorescence. Fluorescent readouts from treatments were normalized to the respective negative control conditions (no treatment) to calculate CXCR4^+^TNBC cell viability.

### Preparation of inhibitors and testing against co-culture spheroids

The CXCR4^+^TNBC:HMF and CXCR4^+^TNBC: CAFs spheroids were treated with a MEK inhibitor, PD0325901 (Selleckchem), and a PI3K inhibitor, PI-103 (Selleckchem). Stock solutions of the inhibitors were prepared at a 50 mM concentration in DMSO and stored at –80°C. The desired inhibitor concentrations for experiments were prepared by serially diluting each stock solution in 1% FBS-containing cell culture medium. After forming co-culture spheroids, the medium exchange was performed as previously described and 30 μl of an inhibitor solution (prepared at 2X desired concentration) was added to spheroids in the last renewal stage, diluting the inhibitor concentrations in half. A column of spheroids was left untreated and only received 30 μl of fresh 1% FBS-containing medium. Spheroids were incubated for 72 hrs and then treatments were renewed by direct addition of 30 μl of fresh inhibitor solutions (prepared at 1X desired concentration). The negative control conditions received 1% FBS-containing medium. The spheroids were incubated for an additional 48 hrs and viable TNBC cells in co-culture spheroids were detected using their endogenous eGFP with a plate reader. Fluorescent readouts from treatments were normalized to their respective negative controls (no treatment) to calculate CXCR4^+^TNBC cell viability. The results obtained from plate reading were further confirmed by fluorescent microscopy (Axio Observer, Zeiss) followed by image analysis (ImageJ, NIH).

## SUPPLEMENTARY MATERIALS FIGURES AND VIDEO




