# Supplementary material for: Three-dimensional tumor model mimics stromal – breast cancer cells signaling
Source: Oncotarget. 2017 Dec 5;9(1):249–67. doi: 10.18632/oncotarget.22922 (PMC5787462; doi:10.18632/oncotarget.22922)
Supplement: Supplementary file 1 [file oncotarget-09-249-s001.pdf]

Three-dimensional tumor model mimics stromal – breast cancer cells signaling

SUPPLEMENTARY MATERIALS

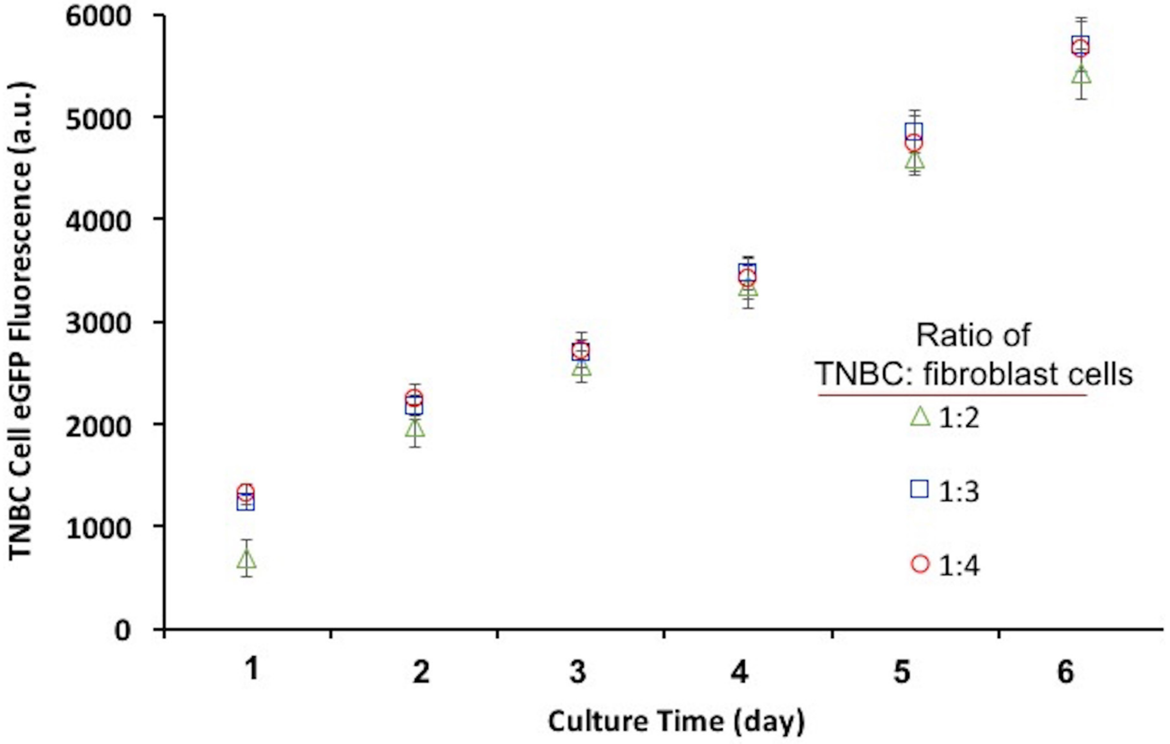

Supplementary Figure 1: Increasing the density of stromal cells in co-culture spheroids to generate ratios of 1:3 and 1:4 (TNBC cells: stromal cells) does not significantly change the breast cancer cells growth compared to the model with 1:2 ratio of TNBC cells: stromal cells.

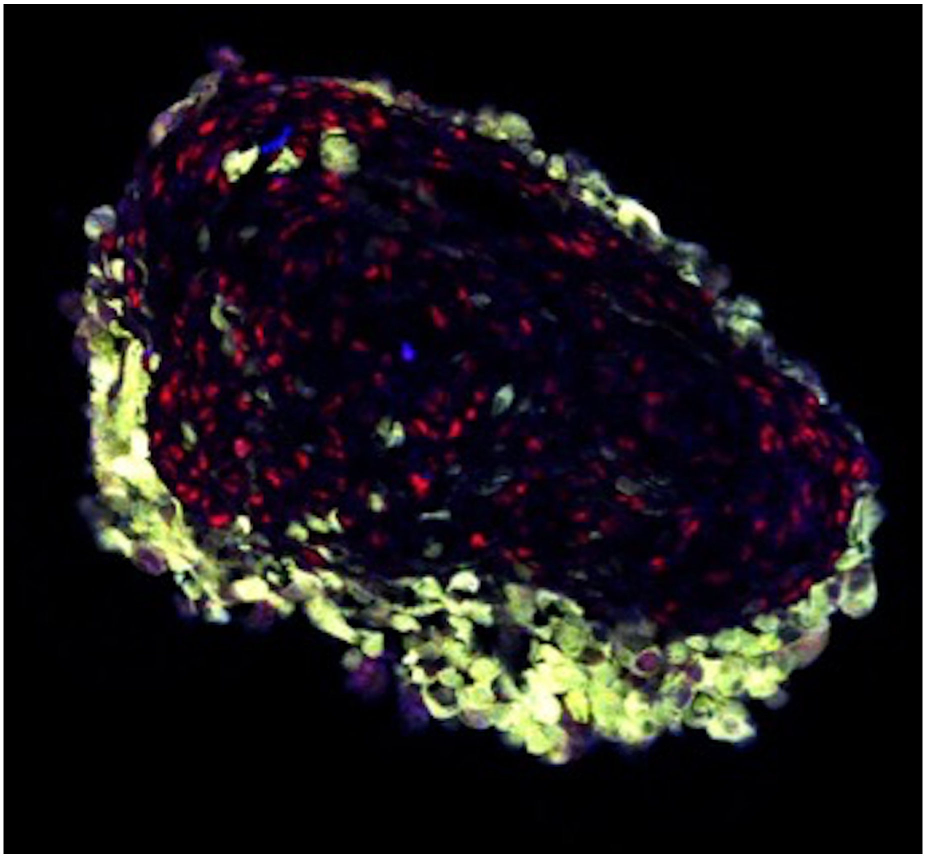

Supplementary Figure 2: A composite image of $CXCR4^+$TNBC:CAFs co-culture model showing that stromal cells are surrounded by the breast cancer cells.

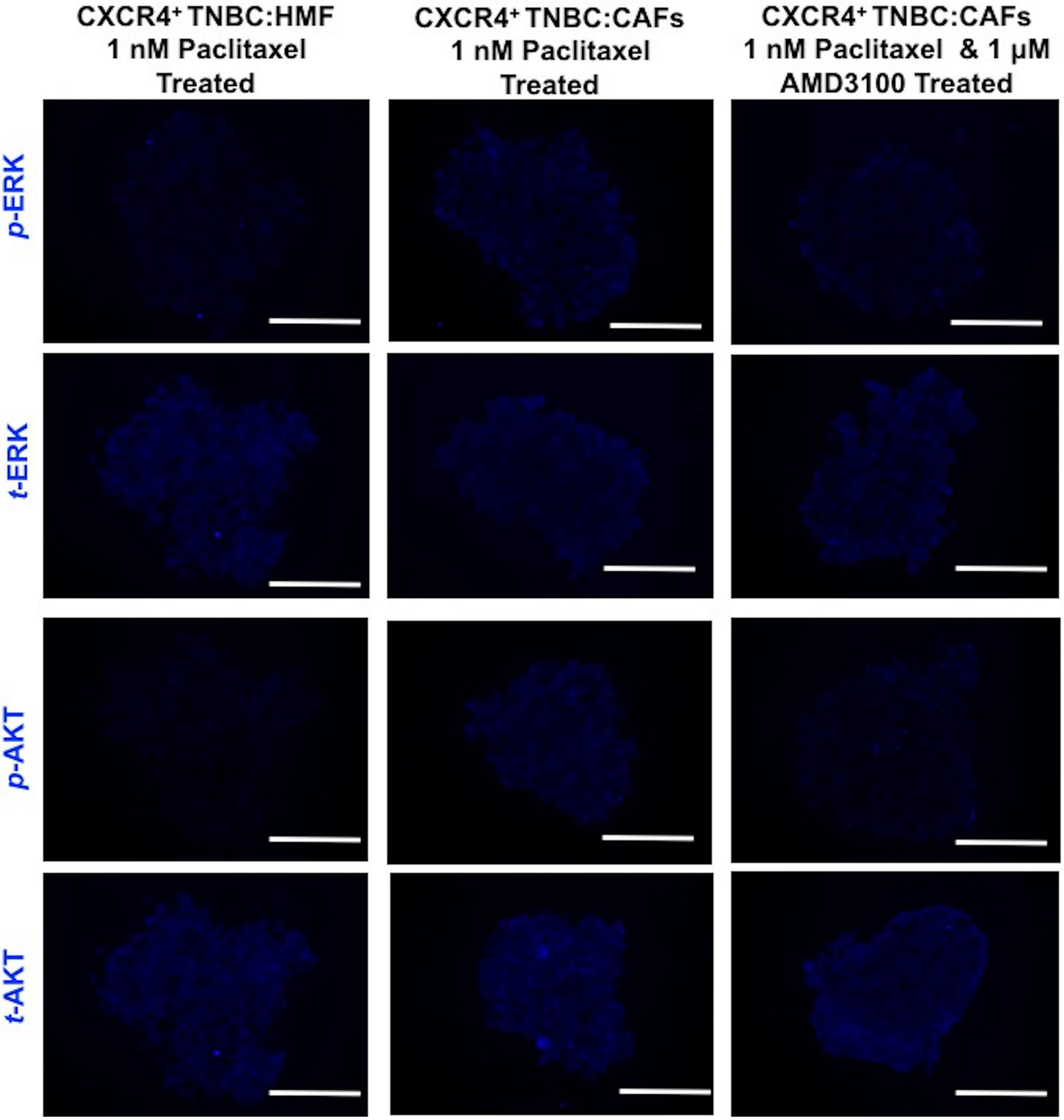

Supplementary Figure 3: Immunohistochemical images corresponding to analyzed data in Figure 9. Cryosections of co-culture spheroids of $CXCR4^{+}$TNBC cells with HMF and CAFs immunostained for ERK and AKT following treatment with 1 nM paclitaxel or combination of 1 nM paclitaxel and 1 μM AMD3100. Scale bar 200 μm.

Supplementary Movie 1: A 3D reconstruction of stromal:TNBC cells spheroid using confocal microscopy shows the distribution of the two cell types. See Supplementary_Movie_1
